# Commentary: Injecting Instructions into Premotor Cortex

**DOI:** 10.3389/fncel.2018.00065

**Published:** 2018-03-27

**Authors:** Mikhail A. Lebedev, Alexei Ossadtchi

**Affiliations:** ^1^Department of Neurobiology, Duke University, Durham, NC, United States; ^2^Center for Bioelectric Interfaces, National Research University Higher School of Economics, Moscow, Russia

**Keywords:** intracortical microstimulation, premotor cortex, monkey, hebbian learning, hebbian plasticity, neural prostheses, predictive coding, predictive processing

Here we call attention to a scholarly paper of particular note, where Mazurek and Schieber (Mazurek and Schieber, 2017) reported for the first time that arm reaching tasks performed by rhesus monkeys can be instructed by intracortical stimulation (ICMS) applied to dorsal premotor cortex (PMd). Monkeys started each trial by grasping with the hand a home handle that was surrounded by four target handles. Next, reach direction was instructed by turning on a display composed of light emitting diodes (LEDs) at the base of the target handle and/or applying ICMS to different sites in PMd. ICMS of the primary somatosensory cortex (S1) was also tested in the same context. Monkeys responded to the instruction by releasing the home handle and grasping the target handle. They learned to respond correctly to both LED and ICMS instructions, with very high success rate (96–99%).

Previously, motor responses have been instructed by ICMS of S1 in owl monkeys (Fitzsimmons et al., 2007), rhesus monkeys (Romo et al., 1998; O'Doherty et al., 2009) and rats (Talwar et al., 2002; Pais-Vieira et al., 2013). In rats, ICMS of M1 has been used for the same purpose (Pais-Vieira et al., 2013). The study of Mazurek and Schieber is innovative because they stimulated a higher-order motor area known to be related to motor preparation (Weinrich and Wise, 1982), visuomotor transformations (Caminiti et al., 1998), nostandard sensorimotor mapping (Wise et al., 1996), but not primary processing of movements or sensations. Therefore, these results could not be readily attributed to ICMS-evoked motor responses (Graziano et al., 2002) or artificial sensations (Romo et al., 1998; Fitzsimmons et al., 2007; O'Doherty et al., 2009).

Mazurek and Schieber kept the amplitude of the ICMS applied to PMd low to make sure that no muscle activations were evoked. While the absence of such activations was confirmed by the pulse-triggered EMG averages, the authors did not illustrate how arm EMGs were modulated during task performance. Such an illustration would be useful because in the video of their experiment, hand movements are visible that occurred before the instruction stimuli and during the reaction time period. Quite surprisingly, lower ICMS currents could be applied to PMd than to S1 to accurately instruct the reach target. Mazurek and Schieber commented, “ICMS thus may be experienced more readily in PM than S1.” Overall, Mazurek and Schieber did not speculate excessively about the nature of experiences evoked by ICMS of PMd but proposed that ICMS “may have evoked somatosensory and/or visual percepts, desires to move particular body parts, or other internal urges or thoughts, any of which the monkeys could have used as instructions.” Percepts in the form of a desire to initiate movement have been reported previously for electrical stimulation of premotor cortex in humans (Penfield and Rasmussen, 1950).

While these results can be generally described as a type of associative learning (Pearce and Bouton, 2001), it is unclear whether monkey's awareness of the experiences evoked by ICMS was essential for such learning. Although Mazurek and Schieber suggest that their monkeys had conscious experiences of ICMS and reported these experiences with arm movements, it is also possible that ICMS induced Hebbian learning (Hebb, 2005) of a nonconscious type (Lewicki et al., 1992; Shanks and John, 1994), where repeated coupling of ICMS with the activation of PMd circuitry during target selection caused specific modifications of synaptic weights for a subset of PMd neurons. Indeed, monkeys were first overtrained on the visually-instructed task. Next, ICMS was repeatedly coupled with the instructions provided by LEDs. Under these conditions, specific populations of PMd neurons were activated while the monkeys responded to each instructed target, and ICMS simultaneously activated axons passing through the stimulated area (Tehovnik et al., 2006). Some of these axons projected to the task-related neurons in PMd, as well task-related neurons in cortical areas interconnected with PMd. Consequently, the effect of Hebbian plasticity was likely to strengthen the responses of specific neuronal populations to ICMS. It is reasonable to suggest that ICMS eventually started triggering decision-related PMd activity in the absence of LED instructions. For such Hebbian plasticity to occur, conscious discrimination of different ICMS patterns is not required. On the other hand, it is possible that Hebbian plasticity contributed to the emergence and shaping of the monkeys' conscious experiences caused by ICMS in this experimental context.

Our view deemphasizes the role of conscious experience, and this is different from the traditional interpretations of ICMS effects. Historically, ICMS has been used for two main purposes: (1) to disrupt cortical processing (Tehovnik and Slocum, 2003; Wegener et al., 2008), and (2) evoke neural responses that mimic functions of the stimulated area (Salzman et al., 1990; Romo et al., 1998; Graziano et al., 2002; Tehovnik et al., 2006; Tehovnik and Slocum, 2007). For the electrical stimulation of cortical sensory areas in humans, such as S1 (Cushing, 1909; Penfield and Boldrey, 1937; Penfield and Rasmussen, 1950; Flesher et al., 2016) and primary visual cortex (Brindley, 1970; Dobelle and Mladejovsky, 1974; Bak et al., 1990), the focus has been traditionally on the perceptions experienced by the subjects. The possibility has received less attention that stimulation may connect to the ongoing cortical activity via a Hebbian mechanism irrespective of the perceptual experience it causes. Yet, several studies have shown that pairing stimulation with motor activity or another stimulus evokes cortical plasticity, such as pairing of transcranial magnetic stimulation in humans with the stimulation of peripheral nerves (Stefan et al., 2000) and artificially connecting two sites in monkey primary motor cortex (Jackson et al., 2006) and S1 (Song et al., 2013). Additionally, cortical plasticity has been demonstrated using cross-modal pairing. For example, Lahav et al. (2007) trained non-musicians to play a piece of music on a piano. Following this training, the sound of music started to activate cortical motor areas even when the subjects did not move their hands. Such cortical plasticity is also consistent with embodied language framework (Pulvermüller, 2013). The fact that subjects can remain unaware of the plastic changes has been elegantly demonstrated using a neurofeedback paradigm (Kaplan et al., 2005). Additionally, it has been shown that training can improve visual sensitivity in blindsight patients (Sahraie et al., 2006; Chokron et al., 2008).

Electrical stimulation of somatosensory system has started to be implemented in bidirectional neural prostheses of the limbs (O'Doherty et al., 2011; Raspopovic et al., 2014; Lebedev and Ossadtchi, 2018). In such systems, Hebbian plasticity could be employed to improve learning of the artificial tactile feedback: electrical stimulation could be paired with virtual reality, tactile stimulation applied to the healthy hand, or verbal stimuli. Such pairing could facilitate the formation of a new percept associated with different sensory modalities and higher-order representations. As pointed out above, stimulation does not necessarily have to mimic the natural activity of the stimulated neuronal circuitry; Hebbian plasticity would eventually make this artificial input more meaningful and possibly consciously perceived. It is possible that Hebbian mechanisms played a role in the previous experiments on ICMS-induced somatosensory perceptions, particularly the ones where training was conducted over the course of many days (Fitzsimmons et al., 2007; O'Doherty et al., 2009, 2011; Tabot et al., 2013). Moreover, the findings of Mazurek and Schieber suggest that the developers of bidirectional neural prostheses could use non-sensory areas as sites for the application of ICMS, and Hebbian associative learning could eventually result in the emergence of realistic perceptions associated with such stimulation.

Finally, ICMS-based neuroprosthetic systems may work optimally if they operate as systems with prediction (Montague and Sejnowski, 1994; Sejnowski et al., 1995; Mirabella and Lebedev, 2017). In such predictive prostheses, ICMS patterns should reflect the properties of prediction error defined as the difference between the internal state and the observed sensory signals. Implementation of such Kalman filtering-based operations (Friston, 2005; Clark, 2015) could incorporate the prosthetic system into the brain more naturally.

## Author contributions

All authors listed have made a substantial, direct and intellectual contribution to the work, and approved it for publication.

### Conflict of interest statement

The authors declare that the research was conducted in the absence of any commercial or financial relationships that could be construed as a potential conflict of interest.

## References

[B1] Bak M. Girvin J. P. Hambrecht F. T. Kufta C. V. Loeb G. E. Schmidt E. M. (1990). Visual sensations produced by intracortical microstimulation of the human occipital cortex. Med. Biol. Eng. Comput. 28, 257–259. 10.1007/BF02442682 2377008

[B2] Brindley G. (1970). Sensations produced by electrical stimulation of the occipital poles of the cerebral hemispheres, and their use in constructing visual prostheses. Ann. R. Coll. Surg. Engl. 47, 106-108. 5452658 PMC2387784

[B3] Caminiti R. Ferraina S. Mayer A. B. (1998). Visuomotor transformations: early cortical mechanisms of reaching. Curr. Opin. Neurobiol. 8, 753–761. 9914239 10.1016/s0959-4388(98)80118-9

[B4] Chokron S. Perez C. Obadia M. Gaudry I. Laloum L. Gout O. (2008). From blindsight to sight: cognitive rehabilitation of visual field defects. Restor. Neuro. Neurosci. 26, 305–320. 18997308

[B5] Clark A. (2015). Surfing Uncertainty: Prediction, Action, and the Embodied Mind. New York, NY: Oxford University Press.

[B6] Cushing H. (1909). A note upon the faradic stimulation of the postcentral gyrus in conscious patients. Brain 32, 44–53. 10.1093/brain/32.1.44

[B7] Dobelle W. H. Mladejovsky M. G. (1974). Phosphenes produced by electrical stimulation of human occipital cortex, and their application to the development of a prosthesis for the blind. J. Physiol. 243, 553–576. 10.1113/jphysiol.1974.sp010766 4449074 PMC1330721

[B8] Fitzsimmons N. A. Drake W. Hanson T. L. Lebedev M. A. Nicolelis M. A. (2007). Primate reaching cued by multichannel spatiotemporal cortical microstimulation. J. Neurosci. 27, 5593–5602. 10.1523/JNEUROSCI.5297-06.2007 17522304 PMC6672750

[B9] Flesher S. N. Collinger J. L. Foldes S. T. Weiss J. M. Downey J. E. Tyler-Kabara E. C.. (2016). Intracortical microstimulation of human somatosensory cortex. Sci. Transl. Med. 8:361ra141 27738096 10.1126/scitranslmed.aaf8083

[B10] Friston K. (2005). A theory of cortical responses. Philos. Trans. R. Soc. Lond. Biol. Sci. 360, 815–836. 10.1098/rstb.2005.1622 15937014 PMC1569488

[B11] Graziano M. S. Taylor C. S. Moore T. (2002). Complex movements evoked by microstimulation of precentral cortex. Neuron 34, 841–851. 10.1016/S0896-6273(02)00698-0 12062029

[B12] Hebb D. O. (2005). The Organization of Behavior: A Neuropsychological Theory. Mahwah, NJ: Psychology Press.

[B13] Jackson A. Mavoori J. Fetz E. E. (2006). Long-term motor cortex plasticity induced by an electronic neural implant. Nature 444, 56–60. 10.1038/nature05226 17057705

[B14] Kaplan A. Y. Byeon J. G. Lim J. J. Jin K. S. Park B. W. Tarasova S. U. (2005). Unconscious operant conditioning in the paradigm of brain-computer interface based on color perception. Int. J. Neurosci. 115, 781–802. 10.1080/00207450590881975 16019574

[B15] Lahav A. Saltzman E. Schlaug G. (2007). Action representation of sound: audiomotor recognition network while listening to newly acquired actions. J. Neurosci. 27, 308–314. 10.1523/JNEUROSCI.4822-06.2007 17215391 PMC6672064

[B16] Lebedev M. A. and Ossadtchi, A. (2018). Bidirectional neural interfaces, in Brain-Computer Interfaces Handbook: Technological and Tpageheoretical Advances, eds Nam C. S. Nijholt A. Lotte F. (Boca Raton, MA; London; New York, NY: CRC Press).

[B17] Lewicki P. Hill T. Czyzewska M. (1992). Nonconscious acquisition of information. Am. Psychol. 47, 796-801. 1616179 10.1037//0003-066x.47.6.796

[B18] Mazurek K. A. Schieber M. H. (2017). Injecting instructions into premotor cortex. Neuron 96, 1282.e4–1289.e4. 10.1016/j.neuron.2017.11.006 29224724 PMC5739962

[B19] Mirabella G. Lebedev M. A. (2017). Interfacing to the brain's motor decisions. J. Neurophysiol. 117, 1305–1319. 10.1152/jn.00051.2016 28003406 PMC5350270

[B20] Montague P. R. Sejnowski T. J. (1994). The predictive brain: temporal coincidence and temporal order in synaptic learning mechanisms. Learn. Mem. 1, 1–33. 10467583

[B21] O'Doherty J. E. Lebedev M. A. Ifft P. J. Zhuang K. Z. Shokur S. Nicolelis H.. (2011). Active tactile exploration using a brain-machine-brain interface. Nature 479, 228–231. 10.1038/nature10489 21976021 PMC3236080

[B22] O'Doherty J. E. Lebedev M. A. Hanson T. L. Fitzsimmons N. A. Nicolelis M. A. (2009). A brain-machine interface instructed by direct intracortical microstimulation. Front. Integr. Neurosci. 3:20. 10.3389/neuro.07.020.2009 19750199 PMC2741294

[B23] Pais-Vieira M. Lebedev M. Kunicki C. Wang J. Nicolelis M. A. (2013). A brain-to-brain interface for real-time sharing of sensorimotor information. Sci. Rep. 3:1319. 10.1038/srep01319 23448946 PMC3584574

[B24] Pearce J. M. Bouton M. E. (2001). Theories of associative learning in animals. Annu. Rev. Psychol. 52, 111–139. 10.1146/annurev.psych.52.1.111 11148301

[B25] Penfield W. Boldrey E. (1937). Somatic motor and sensory representation in the cerebral cortex of man as studied by electrical stimulation. Brain 60, 389–443. 10.1093/brain/60.4.389

[B26] Penfield W. Rasmussen T. (1950). The cerebral cortex of man; a clinical study of localization of function.

[B27] Pulvermüller F. (2013). How neurons make meaning: brain mechanisms for embodied and abstract-symbolic semantics. Trends Cogn. Sci. 17, 458–470. 10.1016/j.tics.2013.06.004 23932069

[B28] Raspopovic S. Capogrosso M. Petrini F. M. Bonizzato M. Rigosa J. Di Pino G.. (2014). Restoring natural sensory feedback in real-time bidirectional hand prostheses. Sci. Transl. Med. 6:222ra19. 10.1126/scitranslmed.3006820 24500407

[B29] Romo R. Hernández A. Zainos A. Salinas E. (1998). Somatosensory discrimination based on cortical microstimulation. Nature 392, 387–390. 10.1038/32891 9537321

[B30] Sahraie A. Trevethan C. T. MacLeod M. J. Murray A. D. Olson J. A. Weiskrantz L. (2006). Increased sensitivity after repeated stimulation of residual spatial channels in blindsight. Proc. Nat. Acad. Sci. U.S.A. 103, 14971–14976. 10.1073/pnas.0607073103 17000999 PMC1595460

[B31] Salzman C. D. Britten K. H. Newsome W. T. (1990). Cortical microstimulation influences perceptual judgements of motion direction. Nature 346, 174–177. 2366872 10.1038/346174a0

[B32] Sejnowski T. J. Dayan P. Montague P. R. (1995). Predictive hebbian learning, in Proceedings of the Eighth Annual Conference on Computational Learning Theory (New York, NY: ACM).

[B33] Shanks D. R. John M. F. S. (1994). Characteristics of dissociable human learning systems. Behav. Brain Sci. 17, 367–395. 10.1017/S0140525X00035032

[B34] Song W. Kerr C. C. Lytton W. W. Francis J. T. (2013). Cortical plasticity induced by spike-triggered microstimulation in primate somatosensory cortex. PLoS ONE 8:e57453. 10.1371/journal.pone.0057453 23472086 PMC3589388

[B35] Stefan K. Kunesch E. Cohen L. G. Benecke R. Classen J. (2000). Induction of plasticity in the human motor cortex by paired associative stimulation. Brain 123, 572–584. 10.1093/brain/123.3.572 10686179

[B36] Tabot G. A. Dammann J. F. Berg J. A. Tenore F. V. Boback J. L. Bensmaia R. J.. (2013). Restoring the sense of touch with a prosthetic hand through a brain interface. Proc. Natl. Acad. Sci. U.S.A. 110, 18279–18284. 10.1073/pnas.1221113110 24127595 PMC3831459

[B37] Talwar S. K. Xu S. Hawley E. S. Weiss S. A. Moxon K. A. Chapin J. K. (2002). Rat navigation guided by remote control. Nature 417, 37–38. 10.1038/417037a 11986657

[B38] Tehovnik E. J. Tolias A. S. Sultan F. Slocum W. M. Logothetis N. K. (2006). Direct and indirect activation of cortical neurons by electrical microstimulation. J. Neurophysiol. 96, 512–521. 10.1152/jn.00126.2006 16835359

[B39] Tehovnik E. J. Slocum W. M. (2003). Microstimulation of macaque V1 disrupts target selection: effects of stimulation polarity. Exp. Brain Res. 148, 233–237. 10.1007/s00221-002-1312-5 12520412

[B40] Tehovnik E. J. Slocum W. M. (2007). Phosphene induction by microstimulation of macaque V1. Brain Res. Rev. 53, 337–343. 10.1016/j.brainresrev.2006.11.001 17173976 PMC1850969

[B41] Wegener S. P. Johnston K. Everling S. (2008). Microstimulation of monkey dorsolateral prefrontal cortex impairs antisaccade performance. Exp. Brain Res. 190, 463–473. 10.1007/s00221-008-1488-4 18641976

[B42] Weinrich M. Wise S. P. (1982). The premotor cortex of the monkey. J. Neurosci. 2, 1329–1345. 7119878 10.1523/JNEUROSCI.02-09-01329.1982 PMC6564318

[B43] Wise S. P. di Pellegrino G. Boussaoud D. (1996). The premotor cortex and nonstandard sensorimotor mapping. Can. J. Physiol. Pharmacol. 74, 469–482. 8828893 10.1139/cjpp-74-4-469

